# Information Theory in Emerging Wireless Communication Systems and Networks

**DOI:** 10.3390/e26070543

**Published:** 2024-06-26

**Authors:** Erdem Koyuncu

**Affiliations:** Department of Electrical and Computer Engineering, University of Illinois Chicago, Chicago, IL 60607, USA; ekoyuncu@uic.edu

## 1. Introduction

Wireless communication systems and networks are rapidly evolving to meet the increasing demands for higher data rates, better reliability, and connectivity anywhere, anytime. There are various new technologies and communication techniques that are currently being proposed for autonomous aerial networks [1,2], the Internet of Space Things [3], terahertz communications [4], large-scale massive multiple-input multiple-output (MIMO) [5,6], intelligent reflecting surfaces (IRSs) [7], visible light communication (VLC) [8], blockchain-based wireless systems [9], and quantum communications and computing [10], just to name a few. Artificial intelligence, machine learning, and edge computing are also expected to be key features of tomorrow’s wireless systems [11,12,13,14]. The nascent nature of the many new proposed methods begs the following fundamental questions: What are the ultimate performance limits of emerging wireless communication systems and networks? How can we design practical schemes to achieve the ultimate limits? Since its inception over 70 years ago by Claude Shannon [15], information theory has been the primary tool for studying the performance limits of communication systems and networks [16]. Consequently, this Special Issue is focused on the information-theoretic analysis of emerging wireless communication systems and networks.

This Special Issue features seven contributions that explore various areas of wireless communications from an information-theoretical perspective. Specifically, two papers focus on full-duplex communications, one on aerial communications, two on intelligent reflecting surfaces, one on polar coding, three on artificial intelligence techniques in wireless communications, and one on wireless system security. Detailed descriptions of these contributions are provided below.

## 2. An Overview of Published Articles

Full-duplex communication has recently become a fundamental technique to meet the continuously increasing rate demands of emerging wireless systems [17]. By allowing communication on the same frequency band simultaneously, the aggregate data rate of the links can effectively be doubled, achieving significant gains in spectral efficiency [18]. The first article of the Special Issue, contribution 1, investigates the power allocation problem in a full-duplex, two-way (FDTW) communication network over an OFDM channel, aiming at improving the sum data rate and energy efficiency. The authors first characterize the sum rate and energy efficiency achieved in a single-carrier FDTW system. The optimal transmit power that achieves the maximal sum data rate is presented. The energy efficiency maximization problem is solved by using fractional programming. The authors then extend their results to a multi-subcarrier FDTW system using an iterative algorithm, achieving a solution that is close to globally optimal. Simulation results show that using the obtained optimal transmit power allocation algorithm can significantly improve the sum rate and energy efficiency in both single-carrier and multi-subcarrier systems.

Intelligent reflecting surfaces (IRSs) and unmanned aerial vehicles (UAVs) are also considered to be key features of future-generation communication systems. IRSs can enhance signal strength and coverage by intelligently tuning signal reflections at a large number of passive elements [7], while UAVs can provide enhanced signal coverage to remote locations and in the presence of geographical obstructions [2]. A UAV equipped with an IRS can provide several benefits in terms of maximizing reliability or rates [19]. In particular, contribution 2 studies IRS-assisted secure transmission in UAV communication systems, where the UAV base station, the legitimate receiver, and the malicious eavesdropper in the system are all equipped with multiple antennas. In order to maximize the secrecy rate, the transmit precoding matrix, the artificial noise matrix, the IRS phase shift matrix, and the UAV’s position are jointly optimized subject to the constraints of the transmit power limit, unit modulus of IRS phase shift, and maximum moving distance of the UAV. Since the problem is non-convex, an alternating optimization (AO) algorithm is proposed to solve it. The author’s theoretical analysis and simulation results show that the proposed AO algorithm has a good convergence performance and can increase the SR by 40.5% compared with the method without IRS assistance.

Contribution 3 investigates two-way communications between an access point (AP) and multiple terminals in low-cost Internet of Things (IoT) networks. The main issues considered are the asymmetric transmission traffic on the uplink (UL) and downlink (DL) and the unbalanced receivers’ processing capability at the AP and the terminals. As a solution, a hybrid non-orthogonal multiple-access/orthogonal multiple-access (NoMA/OMA) scheme together with a joint power and time allocation method is proposed. For the system’s design, the authors formulate the optimization problem with the aim of minimizing the system power and satisfying the UL and DL transmission rate constraints. Due to the coupling of power and time variables in the objective function and the multi-user interference (MUI) in the UL transmission rate constraints, the formulated problem is shown to be non-linear and non-convex and thus is hard to solve. To obtain a numerical, efficient solution, the original problem is first reformulated to be a convex problem relying on the successive convex approximation (SCA) method, and then a numerical efficient solution is thus obtained by using an iterative routine. The proposed transmission scheme is shown to be not only physically feasible but also power-efficient.

As one of the biggest breakthroughs in information theory, polar codes were introduced by Arikan as the first provably capacity-achieving channel code with a low encoding and decoding complexity [20]. Applications include 6G and beyond wireless communication, data compression, and error correction in digital communications. This Special Issue also touches on this fundamental area through contribution 4, which discusses the advancements in polar code decoding. Contribution 4 focuses on cyclic redundancy check (CRC)-aided successive cancellation list (CA-SCL) decoding, which is a powerful algorithm that dramatically improves the error performance of polar codes. Path selection is a major issue that affects the decoding latency of SCL decoders. Generally, it is implemented using a metric sorter, which causes its latency to increase as the list grows. In contribution 4, neural-network-based intelligent path selection (IPS) is proposed as an alternative to the traditional metric sorter. The results show that the proposed path selection method can achieve comparable performance gains to existing methods under SCL/CA-SCL decoding. Compared with the conventional methods, IPS has a lower latency for medium and large list sizes. For the proposed hardware structure, the time complexity of IPS is $O(k\log_{2}L)$, where *k* is the number of hidden layers in the network and *L* is the list size.

The next paper of the Special Issue continues the “AI for Wireless” theme of contribution 4 by applying deep Q-learning (DQL) algorithms to wireless resource allocation problems. Specifically, contribution 5 addresses a downlink resource allocation problem in distributed interference orthogonal frequency-division multiple-access (OFDMA) systems under maximal power constraints. As next-generation wireless networks become increasingly complex and heterogeneous, optimizing system performance metrics while guaranteeing user service requests becomes challenging. Traditional approaches for these non-convex problems are computationally expensive. This paper explores a DQL-based approach to optimize transmit power control for users in multi-cell interference networks, aiming to maximize overall system throughput while meeting maximum power and signal-to-interference-plus-noise ratio (SINR) constraints. The problem is formulated as a non-cooperative game, where base stations (BSs) compete to improve their utility functions while ensuring quality of service (QoS) requirements. Numerical simulations show that the proposed DQL-based scheme outperforms traditional solutions, effectively maximizing system throughput while satisfying power and spectral efficiency requirements.

The next paper of the Special Issue, contribution 6, continues the exploration of IRSs discussed in contribution 2, which combined RISs and UAVs to enhance secure communication. Contribution 6 focuses on the optimal deployment of an IRS element in a line-of-sight domain (LoSD) based on a realistic deployment scenario, which jointly considers the path loss, transmit power, and energy efficiency of the system. Furthermore, the authors aim to minimize the transmit power via jointly optimizing its transmit beamforming and the reflect phase shifts of the IRS subject to the quality-of-service (QoS) constraint, namely, the signal-to-noise ratio (SNR) constraint at the user. However, their optimization problem is non-convex with intricately coupled variables. To tackle this challenge, they apply proper transformations on the QoS constraint and then propose an efficient alternating optimization (AO) algorithm. The simulation results demonstrate that compared to a conventional endpoint deployment strategy that simply deploys an IRS at the transceiver ends, their proposed LoSD deployment strategy significantly reduces the transmit power by optimizing the available LoS links when a single IRS is relayed.

Finally, like contribution 5, contribution 7 of this Special Issue focuses on novel AI applications in wireless systems, studying secure communication design [21]. The communication reliability of wireless communication systems is threatened by malicious jammers. Aiming at the problem of reliable communication under malicious jamming, a large number of schemes have been proposed to mitigate the effects of malicious jamming by avoiding the blocking interference of jammers. However, existing anti-jamming schemes, such as DQNs, use a limited amount of historical data. In view of this, contribution 7 proposes anti-jamming communication using imitation learning. Specifically, the authors propose an algorithm that consists of three steps: First, the heuristic-based expert trajectory generation algorithm is proposed as the expert strategy, which enables the obtention of the expert trajectory from historical samples. The trajectory mentioned in this algorithm represents the sequence of actions undertaken by the expert in various situations. Then, the authors obtain a user strategy by imitating the expert strategy using an imitation learning neural network. Finally, they adopt a functional user strategy for efficient and sequential anti-jamming decisions. The simulation results indicate that the proposed method outperforms DQN-based anti-jamming methods and provides robustness against channel fading, noise, and different jamming patterns.

## 3. Conclusions

This Special Issue presents seven contributions covering a wide range of emerging areas in wireless communications. Current trends indicate that multiple-antenna communications, such as those using continuous reflecting surfaces, along with full-duplex concepts, will remain dominant research directions for next-generation systems. It is also evident that machine learning or “AI for Wireless” methodologies are becoming increasingly common for optimizing wireless communication systems. This shows that AI technologies can potentially become more important in shaping the future of wireless networks.
